# Exposure to Dibutyl Phthalate and Reproductive-Related Outcomes in Animal Models: Evidence From Rodents Study

**DOI:** 10.3389/fphys.2021.684532

**Published:** 2021-12-08

**Authors:** Jiawei Wang, Xi Zhang, Yang Li, Yingqing Liu, Lingsong Tao

**Affiliations:** ^1^Department of Urology, The Second People’s Hospital of Wuhu, Wuhu, China; ^2^The State Key Laboratory of Reproductive Medicine, Department of Urology, The First Affiliated Hospital of Nanjing Medical University, Nanjing, China; ^3^Department of Urology, Shanghai Pudong Hospital, Fudan University Pudong Medical Center, Shanghai, China

**Keywords:** review, animal experimentation, male genitalia, reproductive, DBP

## Abstract

**Background:** Dibutyl phthalate (DBP) was an endocrine disruptor, which may lead to cancer and affects reproductive function when accumulated in the body. But the precise role of DBP in the reproductive system remained controversial.

**Objective:** We employed the meta-analysis to explore the relationship between DBP and reproductive-related outcomes.

**Methods:** We searched relevant literature in PubMed, EMBASE, and Web of Science databases. The standardized mean differences (SMDs) and their 95% CIs were measured by random-effects models. Funnel plots and Egger’s regression test were applied to assess publication bias.

**Results:** Finally, 19 literatures were included in this research. The outcomes revealed that DBP was negatively correlated with reproductive organs weight (testis weight: SMD: −0.59; 95% Cl: −1.23, −0.23; seminal vesicles weight: SMD: −0.74; 95% Cl: −1.21, −0.27; prostate weight: SMD: −0.46; 95% Cl: −0.76, −0.16) and sperm parameters (sperm morphology: SMD: 1.29; 95% Cl: 0.63, 1.94; sperm count: SMD: −1.81; 95% Cl: −2.39, −1.23; sperm motility: SMD: −1.92; 95% Cl: −2.62, −1.23).

**Conclusion:** Our research demonstrated that DBP may be negatively associated with reproductive-related indicators, especially at Gestation exposure period and middle dose (100–500 mg/kg/day).

## Introduction

Recently, many researches have revealed that the decline of male reproductive health and fertility was related to toxic substances found in the environment, particularly endocrine-disrupting chemicals such as phthalates (Foster et al., 1980; Hu et al., 2020; Rodriguez-Sosa et al., 2020; Trovalusci et al., 2020). In addition, dibutyl phthalate (DBP) was also used in building materials, food packaging products, children’s products, and medical equipment all over the world, and it was ubiquitous in nature (Guerra et al., 2010; Man et al., 2010; Jiang et al., 2011). Since DBP may easily leak into food, water, and the ecological environment, it has aroused widespread concern in the scientific community and the public (Chapin et al., 1998; Aurela et al., 1999; Petersen and Breindahl, 2000). Recently, a study stated that urine samples from 289 adult reference populations contained 7 to 294 ng/ml monobutyl phthalate (Petersen and Breindahl, 2000). Hence, because of the widespread existence of DBP, the safe dose of DBP and its harm to the human body have attracted adequate attention. The US Food and Drug Administration (FDA) has set the safe reference dose (RfD) at 0.1 mg/kg/d for humans based on a 1,000-fold reduction of the dose used in the rodent study (Smirnova et al., 2021).

Many studies have employed animal models to explore the function of DBP in the reproductive system and have obtained preliminary results. But, the experimental results were controversial because of differences in animal models, dosages, and methods of administration. In addition, there were controversies as to whether there was a dose-dependent relationship between DBP and reproductive system; whether the exposure days had great relation with the negative outcomes; whether the exposure period in gestation was more harmful than the postnatal period; and so on. For instance, one research, conducted by Martino-Andrade et al. (2009) showed that in the Wistar rat model, a high dose (500 mg/kg/d) of DBP caused an increase in sperm count. However, another research, conducted by Ahmad et al. (2014) revealed that at low doses (50 mg/kg/d) of DBP, the sperm count of Albino rats was significantly reduced.

Therefore, we conducted this study to evaluate the existing animal models more strictly through systematic analysis of the experimental studies that reported the influence of DBP on the reproductive system. These experiment researches reported the relationship between DBP dose, exposure time and exposure period, and reproductive-related outcomes: sperm morphology, sperm count, sperm motility, testis weight, prostate weight, and seminal vesicle weight (Table 1).

**TABLE 1:** PECO statement (population, exposure, comparator and outcomes).

**Variable**	**Description**
Population	Experimental animal studies
Exposure	Exposure to dibutyl phthalate
Comparator	Animals exposed to corn oil treatment
Outcomes	Sperm motility, sperm count, sperm abnormalities, testis weight, seminal vesicle weight, prostate weight

## Methods

This meta-analysis abided by the Systematic Review Centre for Laboratory Animal Experimentation (SYRCLE; Zhang et al., 2020) and PRISMA guidelines (Tekin et al., 2020).

### Search Strategy

From establishment to November 2020, we searched PubMed, EMBASE, and Web of Science databases to include all researches on the impact of DBP on the reproductive system. The search strategy in PubMed, EMBASE, and Web of Science databases contained the fields of Medical Subject Heading (MeSH) terminologies: “dibutyl phthalate,” “genitalia,” “rodentia,” and “spermatozoa.” Meanwhile, we used “AND” and “OR” to combine these terminologies. Supplementary Table 1 showed the synthetically searched strategies.

### Study Selection

The process of screening literature consisted of two steps and was done independently by two researchers at the same time. First, we searched the title and abstract of the article to exclude articles irrelevant to this research. Second, we further searched the full text of the article to include articles that meet the inclusion criteria. When necessary, we resolved differences through discussion.

The inclusive criteria were as follows: (1) laboratory animal research; (2) exposure to DBP; and (3) target outcome indicators (at least one indicator: testis weight, sperm count, seminal vesicle weight, sperm morphology, sperm motility, prostate weight).

The exclusive criteria were as follows: (1) no animals research; (2) vitro experiments; (3) non-randomized controlled trials; (4) no reproductive-related outcomes; (5) exposure to chemicals other than DBP; (6) the experimental subgroup was exposed to the mixture of DBP and other chemicals; and (7) the baseline data of the experimental subgroups and the control subgroups were significantly different.

### Study Characteristics and Data Extraction

We used predesigned data extraction tables to extract data from included studies for evaluation and analysis. All eligible studies recorded the following characteristics and data: (1) the year of publication and the name of the first author; (2) the number and species of animals; (3) the time, dose, and DBP exposure route; (4) exposure days and exposure period; and (5) the result data were expressed by mean ± SD. When the data were missing or ambiguous, we tried to contact the author to obtain the original data.

### Risk of Bias and Methodological Quality Assessment

We used SYRCLE’s risk of bias (RoB) tool to evaluate the risk of bias in the included studies. SYRCLE’s RoB tool could be adjusted for biases that played a particular part in animal experiments (Hooijmans et al., 2014). According to the descriptions of SYRCLE’s RoB tool, the authors (ZX and JW) autonomously evaluated the risk of bias and settled the differences through discussion if necessary. The RoB tool was made up of 10 projects that could assess six forms of biases: selection, performance, reporting, detection, attrition, and other bias (Hooijmans et al., 2014). The detailed information was shown in Supplementary Table 2.

### Statistical Analysis

We used Stata 14.0 software (Stata Corporation, College Station, TX, United States) to analyze the data. The *P*-value was bilateral and if *P* ≥ 0.05, there was no statistical significance. In the meta-analysis, heterogeneity tests were performed for standardized mean differences (SMDs), Cochrane Q test (Lau et al., 1997), and inconsistent index values (I^2^) (Higgins et al., 2003). According to the requirements of nucleoside triphosphate (NTP) 2015 (Nelson et al., 2020), the I^2^ used to measure heterogeneity was considered as no serious heterogeneity, moderate heterogeneity, and substantial heterogeneity at <50, 50–75, and >75%, respectively. At the same time, the subgroup analysis was carried out according to the schematic diagram defined in the PROSPERO scheme to find the logical causes of heterogeneity, including exposure dose, exposure days, and exposure period.

We first determined the publication bias by visually inspecting the symmetry of the funnel chart. However, due to the subjective nature of the visual inspection, we subsequently used Egger’s test for further verification. When the value was greater than 0.1, the *P*-value of Egger’s test was deemed to have no significant publishing bias. In addition, we further investigated the potential conflict of interest sections related to the source of funds. DBP was of great economic and commercial value, and when non-governmental organizations (NGOs) (or industry) sponsored this research, there may be potential publishing bias.

Therefore, we classified the publication bias research with strong suspicion into three situations or a combination of three situations: asymmetric funnel map; lag time of “negative results” researches; and NGO funding or conflict of interest, which would lower the confidence rating. To evaluate the robustness of the consolidated outcomes, we employed a sensitivity analysis. Besides, to exclude studies that lead to significant deviations in the combined effect size, we used Stata software to screen the studies in turn.

## Results

### Features of Included Studies

**FIGURE 1:**
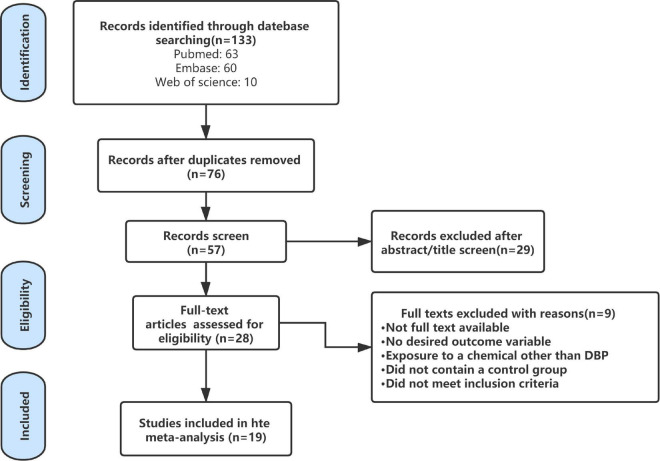
Flow diagram of literature search and selection process.

The flow chart of the literature retrieval and selection process was shown in Figure 1. By combining the search result in EMBASE, Web of Science, and PubMed, a total of 133 studies were initially found, but 76 duplicate studies were excluded. Next, 28 studies were reserved after screening the titles and abstracts. Through the evaluation of the rest of the researches, nine researches, including conference reports, non-human studies, or studies unrelated to the analysis, were excluded. In the end, we included 19 researches for analysis (Tsutsumi et al., 2004; Zhang et al., 2004; Farombi et al., 2007; Hutchison et al., 2008; Kim et al., 2010; Scarano et al., 2010; Bao et al., 2011; Ahmad et al., 2014; Giribabu et al., 2014; Nair, 2015; Aly et al., 2016; Dobrzyńska et al., 2017; Giribabu and Reddy, 2017; Nelli and Pamanji, 2017; Negrin et al., 2018; de Souza et al., 2019; Venturelli et al., 2019; Wang et al., 2019) (Table 2).

**TABLE 2:** Characteristics of enrolled studies.

**References**	**Species (strain)**	**Dose in mg/kg/bw/day**	**Exposure period**	**Exposure route**	**Exposure days**	**Effects on male reproduction extracted in the analysis**
de Souza et al., 2019	Rat (Sprague-Dawley)	500	GD21-PND112	Diet	112	Testis weight
Wang et al., 2019	Rat (Sprague-Dawley)	50/500/1000	PND35-PND70	Diet	35	Sperm count, sperm abnormalities
Venturelli et al., 2019	Rat (Wistar)	5/50/500	GD13-PND21	Gavage	31	Testis weight, Seminal vesicle weight, prostate weight
Negrin et al., 2018	Rat Mongolian gerbils (Meriones unguiculatus)	100	GD8-GD23	Gavage	15	Testis weight, sperm count
Dobrzyńska et al., 2017	Mice (Pzh:Sfis outbred male mice)	500/2000	PND31-PND59 PND31-PND87 PND31-PND115 (3 times a week)	Gavage	28/56/84	Testis weight, sperm count, sperm abnormalities, sperm motility
Giribabu and Reddy, 2017	Rat (Wistar)	100/500	GD1,7,14	Injection	3	sperm abnormalities
Giribabu and Reddy, 2017	Rat (Wistar)	100/500	PND90-PND155	Injection	65	Testis weight, Sperm count, sperm motility, sperm abnormalities, seminal vesicle weight, prostate weight
Aly et al., 2016	Rat (Wistar)	200/400/600	PND97-PND112	Gavage	15	Testis weight, sperm count, sperm motility
Nair, 2015	Rat (Wistar)	500/1000/1500	PND49-PND56	Oral	7	Testis weight
Ahmad et al., 2014	Rat (Albino)	2/10/50	GD14-PND1	Oral	10	Testis weight, sperm count, sperm motility, sperm abnormalities, seminal vesicle weight, prostate weight
Giribabu et al., 2014	Rat (Wistar)	100/500	GD1,7,14	Gavage	3	Testis weight, seminal vesicle weight, prostate weight, sperm count, sperm motility
Bao et al., 2011	Rat (Sprague-Dawley)	0.1/1/10/100/500	PND35-PND65	Gavage	30	Testis weight
Scarano et al., 2010	Rat (Wistar)	100	GD12-PND21	Gavage	32	Testis weight, seminal vesicle weight, prostate weight
Kim et al., 2010	Rat (Sprague-Dawley)	250/500/700	GND10-GND19	Gavage	9	Testis weight, seminal vesicle weight, prostate weight
Martino-Andrade et al., 2009	Rat (Wistar)	100/500	GD13-GD21	Oral	8	Testis weight, seminal vesicle weight, prostate weight
Hutchison et al., 2008	Rat (Wistar)	500	GD13.5-GD21.5	Gavage	8	Testis weight
Farombi et al., 2007	Rat (Wistar)	2000	PND49-PND58	Gavage	9	Testis weight, sperm count, sperm motility, sperm abnormalities, seminal vesicle weight, prostate weight
Tsutsumi et al., 2004	Rat (F344)	60/250/1000	PND77-PND105	Diet	28	Testis weight, sperm motility, sperm abnormalities, sperm count, seminal vesicle weight
Zhang et al., 2004	Rat (Sprague-Dawley)	50/250/500	GD1 – PND21	Gavage	54	Sperm abnormalities, sperm motility, sperm count, prostate weight

### Risk of Bias Evaluation

**FIGURE 2:**
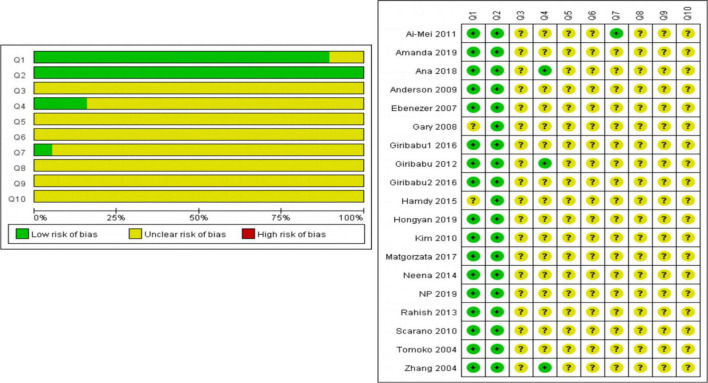
The preliminary analysis results of RoB’s assessment and methodological quality indicators.

The preliminary analysis outcomes of RoB’s assessment were shown in Supplementary Table 2 and Figure 2. Most outstandingly, many items were evaluated as “unclear,” which may cause RoB to be unclear. Regarding selection bias (Q1–Q3), the most enrolled studies (17 of 20) randomly allotted the experimental animals (Q1); all the studies provided the baseline information (most weight, age, or both of them) on experimental animals (Q2); but no study revealed the condition, whether their assignment of different groups was well concealed (Q3). On the spectrum of Q4 and Q5 (performance bias), only three studies stated that the animals were randomly housed during the experiment (Q4) and no study showed that the caregivers and/or investigators were blinded from knowledge which intervention each animal received during the experiment (Q5). For detection bias (Q6 and Q7), no study stated that randomly selected animals were evaluated, yet only one study reported the outcome assessor was blinded. All studies had an unclear risk of attrition bias and reporting bias (Q8 and Q9). There were no other problems in the experiment that could lead to a high risk of bias (Q10).

### Influences of Dibutyl Phthalate on the Reproductive System

Tables 3, 4 provided the overall meta-analysis outcomes of enrolled researches. Complete forest figures were shown in Supplementary Figures 2–7.

**TABLE 3:** The result of meta-analysis in organ weight.

**Analysis**	**Total/subgroups**	**SMD(95%CI)**	**I^2%^**	**P^*a*^**	**No. studies (no.comparisons)**
Testis weight	Total	−0.59 (−0.95, −0.23)	84.2	<0.001	15 (38)
	Low dose (<100 mg/kg/d)	−0.37 (−1.12, 0.39)	88.0	<0.001	4 (9)
	Moderate dose (100–500 mg/kg/d)	−0.72 (−1.23, −0.22)	84.2	<0.001	14 (21)
	High dose (>500 mg/kg/d)	−0.55 (−1.23, 0.14)	74.6	<0.001	5 (8)
	Exposure ≤ 5 days	−0.77 (−1.27, −0.26)	80.6	<0.001	7 (17)
	Exposure >15 days	−0.45 (−0.98, 0.06)	86.2	<0.001	8 (21)
	Exposure period (gestation)	−0.91 (−1.41, −0.41)	79.5	<0.001	9 (17)
	Exposure period (postnatal)	−0.33 (−0.83, 0.16)	85.6	<0.001	6 (21)
Prostate weight	Total	−0.46 (−0.76, −0.16)	70.7	<0.001	9(20)
	Low dose (<100 mg/kg/d)	−0.58 (−1.23, 0.08)	74.9	0.001	3 (6)
	Moderate dose (100–500 mg/kg/d)	−0.29 (−0.57, −0.01)	42.6	0.058	7 (12)
	High dose (>500 mg/kg/d)	−1.17 (−2.23, −0.10)	76.8	0.038	2 (2)
	Exposure ≤15 days	−0.69 (−1.19, −0.19)	75.6	<0.001	5 (11)
	Exposure >15 days	−0.25 (−0.59, 0.08)	56.6	0.018	4 (9)
	Exposure period (gestation)	−0.64 (−1.03, −0.26)	72.7	<0.001	7 (15)
	Exposure period (postnatal)	−0.04 (−0.30, 0.22)	–	–	2 (5)
Seminal vesicles weight	Total	−0.74 (−1.21, −0.27)	83.5	<0.001	7 (17)
	Low dose (<100 mg/kg/d)	−0.28 (−1.03, 0.47)	76.8	0.001	3 (6)
	Moderate dose (100–500 mg/kg/d)	−0.93 (−1.62, −0.24)	85.3	<0.001	6 (9)
	High dose (>500 mg/kg/d)	−1.09 (−1.54, −0.64)	–	–	2 (2)
	Exposure ≤15 days	−1.25 (−1.85, −0.64)	80.0	<0.001	4 (10)
	Exposure >15 days	0.08 (−0.38, 0.53)	59.8	0.021	3 (7)
	Exposure period (gestation)	−1.11 (−1.68, −0.54)	79.7	<0.001	5 (11)
	Exposure period (postnatal)	0.04 (−0.48, 0.57)	66.3	0.011	2 (6)

*Effect sizes are expressed as the SMD with 95% CIs calculated using random-effects or fixed-effects models. Positive SMDs represent an increase in the outcome measure after exposure. Negative SMDs represent a decrease in the outcome measure after exposure.*

*P^*a*^ is a measure of heterogeneity. *P* > 0.1; Random-effects model was used when *p* value for heterogeneity test <0.1; otherwise, fixed-effects model was used.*

**TABLE 4:** The result of meta-analysis in sperm parameters.

**Analysis**	**Total/subgroups**	**SMD(95%CI)**	**I^2%^**	**P^*a*^**	**No. studies (no.comparisons)**
Sperm count	Total	−1.21 (−2.39, −1.23)	79.4	<0.001	10 (25)
	Low dose (<100 mg/kg/d)	−1.62 (−2.92, −0.32)	76.5	0.002	3 (5)
	Moderate dose (100–500 mg/kg/d)	−2.41 (−3.41, −1.40)	85.0	<0.001	8 (13)
	High dose (>500 mg/kg/d)	−0.95 (−1.54, −0.36)	43.2	0.103	5 (7)
	Exposure ≤15 days	−3.02 (−3.93, −2.10)	70.6	<0.001	5 (10)
	Exposure >15 days	−1.03(−1.63, −0.44)	72.0	<0.001	5(15)
	Exposure period (gestation)	−3.24 (−4.54, −1.95)	79.5	<0.001	4 (7)
	Exposure period (postnatal)	−1.28 (−1.85, −0.70)	73.2	<0.001	6 (18)
Sperm motility	Total	−1.92 (−2.62, −1.23)	78.4	<0.001	7 (19)
	Low dose (<100 mg/kg/d)	−1.69 (−3.29, −0.08)	78.7	0.003	2 (4)
	Moderate dose (100–500 mg/kg/d)	−1.96 (−2.85, −1.07)	75.9	<0.001	5 (10)
	High dose (>500 mg/kg/d)	−2.15 (−3.94, −0.35)	87.1	<0.001	4 (5)
	Exposure ≤ 5 days	−2.93 (−3.95, −1.92)	61.5	0.016	3 (7)
	Exposure >15 days	−1.34 (−2.12, −0.57)	76.8	<0.001	4 (12)
	Exposure period (gestation)	−3.58 (−4.99, −2.16)	74.8	0.001	3 (6)
	Exposure period (postnatal)	−1.24 (−1.87, −0.61)	67.6	<0.001	4 (13)
Sperm abnormality	Total	1.29 (0.63, 1.94)	83.0	<0.001	5 (16)
	Low dose (<100 mg/kg/d)	1.09 (0.18, 2.00)	66.7	0.017	3 (5)
	Moderate dose (100–500 mg/kg/d)	1.80 (0.42, 3.19)	91.2	<0.001	4 (8)
	High dose (>500 mg/kg/d)	1.09 (0.4, 1.79)	41.5	0.144	3 (5)
	Exposure ≤15 days	3.37 (1.74, 5.01)	83.1	<0.001	3 (6)
	Exposure >15 days	0.59 (0.00, 1.17)	75.1	<0.001	3 (12)
	Exposure period (gestation)	2.45 (1.12, 3.78)	91.2	<0.001	3 (8)
	Exposure period (postnatal)	0.64 (0.10, 1.17)	51.3	0.030	3 (10)

*Effect sizes are expressed as the SMD with 95% CIs calculated using random-effects or fixed-effects models. Positive SMDs represent an increase in the outcome measure after exposure. Negative SMDs represent a decrease in the outcome measure after exposure.*

#### Sperm Parameters

In this article, we analyzed the impact of DBP on sperm count, sperm morphology, and sperm motility (Table 3). The results showed that DBP had significantly negative correlation with sperm count (SMD: −1.81; 95% Cl: −2.39, −1.23). In addition, in the DBP exposure period grouping, gestation showed more severe sperm damage (SMD: −3.24; 95%Cl: −4.54, −1.96) compared with postnatal (SMD: −1.28; 95%Cl:− 1.85, −0.70). Interestingly, the shorter the exposure days, the greater the damage to sperm by DBP. Besides, regardless of dosage, sperm count decreased and the middle dose subgroup (SMD: −2.41; 95% Cl: −3.41, −1.40) showed the most negative effects than the low-dose and high-dose groups. Additionally, the outcomes revealed that DBP was related to the reduction of sperm motility (SMD: −1.92; 95% Cl: −2.62, −1.23), especially in the middle dose subgroup (SMD: −1.96; 95% Cl: −2.85, −1.07), gestation subgroup (SMD: −3.58; 95% Cl: −4.99, −2.16), and exposure day ≤15 subgroup (SMD: −2.93; 95% Cl: −3.95, −1.92). Furthermore, the results showed that DBP had negative influence on sperm morphology (SMD: 1.29; 95% Cl: 0.63, 1.94), and the negative influence was more significant in the subgroup of gestation (SMD: 2.45; 95% Cl: 1.12, 3.78), middle dose (SMD: 1.80; 95% Cl: 0.42, 3.19), and exposure days ≤15 (SMD: 3.37; 95% Cl: 1.74, 5.01).

#### Organ Weights

Among the 19 included researches, 16 researches included the relationship between DBP and reproductive organ weight, including testis weight, prostate weight, and seminal vesicle weight (Table 4). In the three subgroups, low dose (<100 mg/kg/d), postnatal, and exposure days (>15 days), we did not observe a significant correlation between DBP and reproductive organ weight. In view of the endocrine disrupting effects of DBP, this result exceeded our expectations. For testis weight (SMD: −0.59; 95% Cl: −1.23, −0.23), the negative influence of subgroups, including gestation group (SMD: −0.91; 95% Cl: −1.41, −0.41) and exposure days (≤15 days) group (SMD: −0.77; 95% Cl: −1.27, −0.26), was greater than that of the opposite subgroups. Besides, the middle dose (100–500 mg/kg/d) had the most negative effects in the dosages. Similarly, on the aspect of seminal vesicle weight (SMD: −0.74; 95% Cl: −1.21, −0.27), subgroup analysis revealed that the seminal vesicle weight decreased more significantly in the exposure days ≤15 subgroup (SMD: −1.25; 95% Cl: −1.85, −0.64), gestation subgroup (SMD: −1.11; 95% Cl: −1.68, −0.54), and middle dose subgroup (100–500 mg/kg/d) (SMD: −0.93; 95% Cl: −1.62, −0.24). Besides, in terms of prostate weight (SMD: −0.46; 95% Cl: −0.76, −0.16), it seemed that the gestation subgroup (SMD: −0.64; 95% Cl: −1.03, −0.26) had more negative effects on prostate weight than the postnatal subgroup (SMD: −0.04; 95% Cl: −0.30, 0.22). Meanwhile, the high dose subgroup (>500 mg/kg/d) (SMD: −1.17; 95% Cl: −2.23, −0.10) had the most negative impact on prostate weight.

### Publication Bias

Supplementary Figure 1 (Funnel plots) and Supplementary Table 3 (Egger’s test) presented the result of publication bias, which indicated notably publication bias in sperm count, sperm morphology, sperm motility, testis weight, and seminal vesicle weight.

Next, we processed the above asymmetric funnel diagram by the trim and filling method, and the results showed that only sperm morphology can eliminate publication bias by the trim and filling method. As shown in Supplementary Figure 8, we need to continue to include three documents with similar results that are similar to those of authors Zhang et al. (2004), Farombi et al. (2007), Ahmad et al. (2014), and Giribabu (2016) to ensure the symmetry of the funnel chart and eliminate publication bias.

### Sensitivity Analyses

Sensitivity analysis examined the impact of each study by ignoring one study at a time and repeating the meta-analysis. Supplementary Table 4 exhibited the result, and there was no change in the associative direction by sensitivity analysis.

## Discussion

Dibutyl phthalate was a common chemical substance, which may affect developmental plasticity and damage reproductive organ weight such as the testis and seminal vesicle (Jiang et al., 2017; Barakat et al., 2019; Chen et al., 2020). As a matter of fact, it has been widely studied that DBP affected spermatogenesis and reproductive organ weight through endocrine disruption (Moody et al., 2014; Gardner et al., 2016; Pan et al., 2017; Rashad et al., 2018). However, the role of DBP in different exposure periods, different exposure days, and the safe dose was still controversial.

Compared with a single study, the meta-analysis may give more dependable outcomes and a vigorous implementation for interpreting controversial conclusions. Therefore, we conducted the research to confirm whether DBP had a significant influence on the reproductive system in different subgroups. To our knowledge, this meta-analysis was the first study to attend to concentrate on the influence of the safety dose, exposure days, and exposure period of DBP on the reproductive system in animals. In this meta-analysis, we discovered that the middle dosages of DBP and gestation exposure period may cause damage to the function of the reproductive system, especially in sperm count, sperm motility, percentage of sperm morphology, testis weight, seminal vesicle weight, and prostate weight.

Our results showed that DBP had a strong association with sperm parameters. To be honest, the negative effects of DBP on sperm parameters have been widely concerned and explored. However, the mechanism of DBP on sperm has not been clearly studied, but the following mechanisms may be mentioned. On the one hand, DBP had an endocrine-disrupting effect, which leads to a significant decrease in testosterone. The decrease of testosterone level may lead to the interruption of the connection between germ cells and sertoli cells, resulting in the decrease of live sperm (Chuang et al., 2020). On the other hand, DBP may penetrate the blood-testis barrier, harm the Leydig cells and sertoli cells, interfere with sperm growth and development, and eventually lead to increased sperm aberration rate (Campbell et al., 2020). Last but not the least, the activity of γ-glutamyl transpeptidase (γ-GT) in the testis of DBP-treated rats was significantly increased. γ-GT was a marker enzyme of sertoli cell function. The activity of this enzyme was inversely proportional to the number and maturity of spermatozoa, which may be related to the change of the function of this enzyme in the maturation and development of germ cells and spermatozoa (Bell et al., 2020). As a matter of fact, from the subgroup analysis, we found that the negative effects of DBP exposure from gestation were more serious than those from postnatal, which may be explained that in the process of fetal gonadal differentiation, neonatal testicular development, and the final maturation and differentiation of adolescent testes, androgen levels had the most significant influence on fetal gonadal differentiation (Dobrzyńska et al., 2017). What is more, the low dose of DBP had no significant effect on the weight of reproductive organs, but it was negatively correlated with sperm parameters, which was inconsistent with the safe dose. Besides, the middle dose had a more significant influence than the high dose, but the specific reason was still unclear. It may be due to the limited literature to explore the high dose. Therefore, more studies were needed to prove the safe dose and explore the effect of high dose on reproduction.

In terms of testis weight, prostate weight, and seminal vesicle weight, DBP might decrease the weight of reproductive organs. From the subgroup analysis, our data suggested that the exposure period gestation had stronger effects on organs weight, but the exposure period postnatal had no significant relation, which may be because low-dose DBP had a “stimulating effect” on postnatal rats (Bao et al., 2011). On the one hand, low-dose DBP had a stimulating effect on serum estradiol (E2), luteinizing hormone (LH), and follicle stimulating hormone (FSH). E2 in the testis can be converted to T by aromatase, and the activity of aromatase was regulated by FSH and LH (Dorrington et al., 1978; Canick et al., 1979). Therefore, the increase in T may be due to the multilevel crosstalk between T, E2, LH, and FSH. On the other hand, low-dose DBP will increase the expression of SOD1 [Cu/Zn superoxide dismutase (SOD)], which reflected the relatively mild oxidative stress experienced by Leydig cells (Luo et al., 2006). In addition, SOD1 may act as a pivotal part in cell proliferation (Takemura et al., 2006) and biosynthesis of steroids (such as T) (Sasaki et al., 1994). But, high-dose DBP exhibited testicular toxicity to postnatal rats and restricted the development of reproductive organs by suppressing the level of T. Thus, when combined with high-dose and low-dose DBP studies, their opposite effects may be neutralized. Besides, the most significant impact of DBP on sertoli cell proliferation/number and testosterone levels in the testis occurred in the late pregnancy (Scott et al., 2008). When male rats are exposed in the uterus, DBP acted as an anti-androgen by reducing the production of fetal testosterone, thereby interfering with the development of reproductive organs.

Our data results showed that DBP had a strong correlation with sperm parameters and reproductive organ development. However, these findings may be limited to a certain extent by the dose of the DBP, majority of the studies we participated in this meta-analysis measured results of ≥100 mg/kg/d, which may be a node with a greater damaging effect. In fact, the outcomes of the subgroup analysis revealed that the greater the dose, the greater the harm of DBP to the reproductive system. This limitation meant that when it came to issues such as the safe dose of DBP and the hazards of small doses, it was necessary to carefully interpret the research results. We believed that more researches on this aspect were necessary.

Finally, although we were rigorously abided by the guiding principle of SRYCLE and obtained reliable statistical evidence of this study, there was still some faultiness that needed to be noted. First, most of the studies we analyzed had unknown RoBs because various projects were rated as “unclear” in terms of evaluation and methodological quality. Meanwhile, there were some gaps in the timing of data collection. In reality, it was common in most animal experiments. In order to correct such conditions, researchers may utilize a detailed list to improve the quality of the experiment in the future, such as the ARRIVE guidelines et al. (Kilkenny et al., 2012). Second, the heterogeneity of this study was inevitable, but subgroup analysis did not effectively reduce heterogeneity, suggesting that the heterogeneity may be related to the variety of experimental design and quality. Hence, the interpretation of the results should be cautious. Third, due to the limitation of data, there was no direct evidence of harm like pathology. In addition, there were few studies with doses lower than 100 mg/kg/d, which may lead to significant deviation and reduce confidence. Therefore, if there were more direct and accurate experiments in the future, the outcomes would be more precise.

## Conclusion

In conclusion, this systematic review helped us to understand the reproductive toxicity of DBP exposure. The results of this paper showed that DBP had a significant negative effect on the weight of testis, epididymis, and seminal vesicles. Second, it was mainly found that it harmed the sperm parameters: sperm motility, sperm morphology, and sperm count. In addition, lower than the safe dose of DBP still showed negative effects on reproductive system outcomes. All in all, our research can also be used to help scholars more fully understand the role of DBP in the reproductive system and we believe that in the future, more studies will pay close attention to the role of DBP, so that the understanding of DBP toxicity will be clearer.

## Data Availability Statement

The raw data supporting the conclusions of this article will be made available by the authors, without undue reservation.

## Author Contributions

YQL and LT developed the statistical method. JW and XZ performed statistical analysis. YL conceived and planned the experiments. JW, YL, and XZ contributed to the interpretation of the results. All authors provided critical feedback and helped shape the research, analysis, and manuscript. All authors read and approved the final manuscript.

## Conflict of Interest

The authors declare that the research was conducted in the absence of any commercial or financial relationships that could be construed as a potential conflict of interest.

## Publisher’s Note

All claims expressed in this article are solely those of the authors and do not necessarily represent those of their affiliated organizations, or those of the publisher, the editors and the reviewers. Any product that may be evaluated in this article, or claim that may be made by its manufacturer, is not guaranteed or endorsed by the publisher.

## References

[B1] Ahmad R. Gautam A. K. Verma Y. Sedha S. Kumar S. (2014). Effects of in utero di-butyl phthalate and butyl benzyl phthalate exposure on offspring development and male reproduction of rat. *Environ. Sci. Pollut. Res. Int.* 21 3156–3165. 10.1007/s11356-013-2281-x 24213843

[B2] Aly H. A. Hassan M. H. El-Beshbishy H. A. Alahdal A. M. Osman A. M. (2016). Dibutyl phthalate induces oxidative stress and impairs spermatogenesis in adult rats. *Toxicol. Ind. Health* 32 1467–1477. 10.1177/0748233714566877 25614580

[B3] Aurela B. Kulmala H. Söderhjelm L. (1999). Phthalates in paper and board packaging and their migration into Tenax and sugar. *Food Addit. Contam.* 16 571–577. 10.1080/026520399283713 10789379

[B4] Bao A. M. Man X. M. Guo X. J. Dong H. B. Wang F. Q. Sun H. (2011). Effects of di-n-butyl phthalate on male rat reproduction following pubertal exposure. *Asian J. Androl.* 13 702–709. 21841806 10.1038/aja.2011.76 PMC3739579

[B5] Barakat R. Seymore T. Lin P. P. Park C. J. Ko C. J. (2019). Prenatal exposure to an environmentally relevant phthalate mixture disrupts testicular steroidogenesis in adult male mice. *Environ. Res.* 172 194–201. 10.1016/j.envres.2019.02.017 30802670 PMC6511329

[B6] Bell S. Zsom A. Conley J. Spade D. (2020). Automated identification of multinucleated germ cells with U-Net. *PLoS One* 15:e0229967. 10.1371/journal.pone.0229967 32645012 PMC7347116

[B7] Campbell J. L. Jr. Otter R. Anderson W. A. Longnecker M. P. Clewell R. A. North C. (2020). Development of a physiologically based pharmacokinetic model of diisononyl phthalate (DiNP) in pregnant rat and human. *J. Toxicol. Environ. Health A.* 83 631–648. 10.1080/15287394.2020.1798831 32757748

[B8] Canick J. A. Makris A. Gunsalus G. L. Ryan K. J. (1979). Testicular aromatization in immature rats: localization and stimulation after gonadotropin administration in vivo. *Endocrinology* 104 285–288. 10.1210/endo-104-2-285 571793

[B9] Chapin R. E. Sloane R. A. Haseman J. K. (1998). Reproductive endpoints in general toxicity studies: are they predictive? *Reprod. Toxicol.* 12 489–494. 9717700 10.1016/s0890-6238(98)00026-4

[B10] Chen H. Chen K. Qiu X. Xu H. Mao G. Zhao T. (2020). The reproductive toxicity and potential mechanisms of combined exposure to dibutyl phthalate and diisobutyl phthalate in male zebrafish (Danio rerio). *Chemosphere* 258:127238. 10.1016/j.chemosphere.2020.127238 32563064

[B11] Chuang S.-C. Chen H.-C. Sun C.-W. Chen Y.-A. Wang Y.-H. Chiang C.-J. (2020). Phthalate exposure and prostate cancer in a population-based nested case-control study. *Environ. Res.* 181:108902. 10.1016/j.envres.2019.108902 31785779

[B12] de Souza N. P. Cardoso A.P. Ferragut Gomide L. M. M. Lima T. R. R. Miot H. A. Martino-Andrade A. J. (2019). Experimental cryptorchidism enhances testicular susceptibility to dibutyl phthalate or acrylamide in Sprague-Dawley rats. *Hum. Exp. Toxicol.* 38 899–913. 30995857 10.1177/0960327119845040

[B13] Dobrzyńska M. M. Tyrkiel E. J. Gajowik A. (2017). Three generation study of reproductive and developmental toxicity following exposure of pubescent F0 male mice to di-n-butyl phthalate. *Mutagenesis* 32 445–454. 10.1093/mutage/gex011 28472404

[B14] Dorrington J. H. Fritz I. B. Armstrong D. T. (1978). Control of testicular estrogen synthesis. *Biol. Reprod.* 18 55–64. 10.1095/biolreprod18.1.55 626767

[B15] Farombi E. O. Abarikwu S. O. Adedara I. A. Oyeyemi M. O. (2007). Curcumin and kolaviron ameliorate di-n-butylphthalate-induced testicular damage in rats. *Basic Clin. Pharmacol. Toxicol.* 100 43–48. 10.1111/j.1742-7843.2007.00005.x 17214610

[B16] Foster P. M. Thomas L. V. Cook M. W. Gangolli S. D. (1980). Study of the testicular effects and changes in zinc excretion produced by some n-alkyl phthalates in the rat. *Toxicol. Appl. Pharmacol.* 54 392–398. 10.1016/0041-008x(80)90165-9 7394794

[B17] Gardner S. T. Wood A. T. Lester R. Onkst P. E. Burnham N. Perygin D. H. (2016). Assessing differences in toxicity and teratogenicity of three phthalates, Diethyl phthalate, Di-n-propyl phthalate, and Di-n-butyl phthalate, using Xenopus laevis embryos. *J. Toxicol. Environ. Health A.* 79 71–82. 10.1080/15287394.2015.1106994 26730679

[B18] Giribabu N. Reddy P. S. (2017). Protection of male reproductive toxicity in rats exposed to di-n-butyl phthalate during embryonic development by testosterone. *Biomed. Pharmacother.* 87 355–365. 10.1016/j.biopha.2016.12.106 28064108

[B19] Giribabu N. Sainath S. B. Reddy P. S. (2014). Prenatal di-n-butyl phthalate exposure alters reproductive functions at adulthood in male rats. *Environ. Toxicol.* 29 534–544. 22489061 10.1002/tox.21779

[B20] Guerra M. T. Scarano W. R. de Toledo F. C. Franci J. A. A. Kempinas W. D. G. (2010). Reproductive development and function of female rats exposed to di-eta-butyl-phthalate (DBP) in utero and during lactation. *Reprod. Toxicol.* 29 99–105. 10.1016/j.reprotox.2009.10.005 19850123

[B21] Higgins J. P. Thompson S. G. Deeks J. J. Altman D. G. (2003). Measuring inconsistency in meta-analyses. *BMJ* 327 557–560. 10.1136/bmj.327.7414.557 12958120 PMC192859

[B22] Hooijmans C. R. Rovers M. M. de Vries R. B. Leenaars M. Ritskes-Hoitinga M. Langendam M. W. (2014). SYRCLE’s risk of bias tool for animal studies. *BMC Med. Res. Methodol.* 14:43. 10.1186/1471-2288-14-43 24667063 PMC4230647

[B23] Hu J. Jiang K. Tang X. Liu H. Zhang H. Yang X. (2020). Chronic exposure to di-n-butyl phthalate causes reproductive toxicity in zebrafish. *J. Appl. Toxicol.* 40 1694–1703. 10.1002/jat.4030 32627227

[B24] Hutchison G. R. Scott H. M. Walker M. McKinnell C. Ferrara D. Mahood I. K. (2008). Sertoli cell development and function in an animal model of testicular dysgenesis syndrome. *Biol. Reprod.* 78 352–360. 10.1095/biolreprod.107.064006 17928633

[B25] Jiang J. T. Sun W. L. Jing Y. F. Liu S. B. Ma Z. Hong Y. (2011). Prenatal exposure to di-n-butyl phthalate induces anorectal malformations in male rat offspring. *Toxicology* 290 323–327. 10.1016/j.tox.2011.10.008 22027561

[B26] Jiang X. P. Tang J. Y. Xu Z. Han P. Qin Z. Q. Yang C. D. (2017). Sulforaphane attenuates di-N-butylphthalate-induced reproductive damage in pubertal mice: Involvement of the Nrf2-antioxidant system. *Environ. Toxicol.* 32 1908–1917. 10.1002/tox.22413 28295950

[B27] Kilkenny C. Browne W. J. Cuthi I. Emerson M. Altman D. G. (2012). Improving bioscience research reporting: the ARRIVE guidelines for reporting animal research. *Vet. Clin. Pathol.* 41 27–31. 10.1111/j.1939-165x.2012.00418.x 22390425

[B28] Kim T. S. Jung K. K. Kim S. S. Kang I. H. Baek J. H. Nam H. S. (2010). Effects of in utero exposure to di(n-butyl) phthalate on development of male reproductive tracts in Sprague-Dawley rats. *J. Toxicol. Environ. Health A.* 73 1544–1559. 10.1080/15287394.2010.511579 20954080

[B29] Lau J. Ioannidis J. P. Schmid C. H. (1997). Quantitative synthesis in systematic reviews. *Ann. Intern. Med.* 127 820–826. 10.7326/0003-4819-127-9-199711010-00008 9382404

[B30] Luo L. Chen H. Trush M. A. Show M. D. Anway M. D. Zirkin B. R. (2006). Aging and the brown Norway rat leydig cell antioxidant defense system. *J. Androl.* 27 240–247. 10.2164/jandrol.05075 16304208

[B31] Man X. M. Qin H. Chen M. J. Zhang C. X. Song L. Wang Y. B. (2010). [Effects of di-butyl phthalate on the reproductive system of adolescent male rats]. *Zhonghua Nan Ke Xue* 16 973–978. 21218637

[B32] Martino-Andrade A. J. Morais R. N. Botelho G. G. K. Muller G. Grande S. W. Carpentieri G. B. (2009). Coadministration of active phthalates results in disruption of foetal testicular function in rats. *Int. J. Androl.* 32 704–712. 10.1111/j.1365-2605.2008.00939.x 19207615

[B33] Moody S. Goh H. Johnson R. Sims N. Loveland K. Itman C. (2014). Chronic exposure to low doses of di-n-butyl phthalate (DBP) results in smaller testes, abnormal testosterone levels, impaired bone health and greater weight gain in adult mice. *Andrology* 2:55.

[B34] Nair N. (2015). Dose-dependent short-term study of di-n-butyl phthalate on the testicular antioxidant system of Wistar rats. *Environ. Sci. Pollut. Res. Int.* 22 2196–2204. 10.1007/s11356-014-3457-8 25172463

[B35] Negrin A. C. de Jesus M. M. Christante C. M. da Silva D. G. H. Taboga S.R. Pinto-Fochi M. E. (2018). Maternal supplementation with corn oil associated or not with di-n-butyl phthalate increases circulating estradiol levels of gerbil offspring and impairs sperm reserve. *Reprod. Toxicol.* 81 168–179. 10.1016/j.reprotox.2018.08.011 30103012

[B36] Nelli G. Pamanji S. R. (2017). Di-n-butyl phthalate prompts interruption of spermatogenesis, steroidogenesis, and fertility associated with increased testicular oxidative stress in adult male rats. *Environ. Sci. Pollut. Res. Int.* 24 18563–18574. 10.1007/s11356-017-9478-3 28646317

[B37] Nelson W. Wang Y.-X. Sakwari G. Ding Y.-B. (2020). Review of the effects of perinatal exposure to endocrine-disrupting chemicals in animals and humans. *Rev. Environ. Contam. Toxicol.* 251 131–184. Editor. 10.1007/398_2019_30 31129734

[B38] Pan Y. Wang X. Yeung L. W. Y. Sheng N. Cui Q. Cui R. (2017). Dietary exposure to di-isobutyl phthalate increases urinary 5-methyl-2′-deoxycytidine level and affects reproductive function in adult male mice. *J. Environ. Sci. (China)* 61 14–23. 10.1016/j.jes.2017.04.036 29191310

[B39] Petersen J. H. Breindahl T. (2000). Plasticizers in total diet samples, baby food and infant formulae. *Food Addit. Contam.* 17 133–141. 10.1080/026520300283487 10793844

[B40] Rashad M. M. Galal M. K. El-Behairy A. M. Gouda E. M. Moussa S. Z. (2018). Maternal exposure to di-n-butyl phthalate induces alterations of c-Myc gene, some apoptotic and growth related genes in pups’ testes. *Toxicol. Ind. Health* 34 744–752. 10.1177/0748233718791623 30231772

[B41] Rodriguez-Sosa J. Ruiz S. Valdez D. Tullot T. (2020). Dibutyl phthalate affects the recovery, size, and viability of pig testicular tissue ectopically grafted in immunocompromised mice. *FASEB J.* 34 1–1. 10.1096/fasebj.2020.34.s1.07518

[B42] Sasaki J. Sato E. F. Nomura T. Mori H. Watanabe S. Kanda S. (1994). Detection of manganese superoxide dismutase mRNA in the theca interna cells of rat ovary during the ovulatory process by in situ hybridization. *Histochemistry* 102 173–176. 10.1007/BF00268893 7868359

[B43] Scarano W. R. Toledo F. C. Guerra M. T. Pinheiro P. F. F. Domeniconi R. F. Felisbino S. L. (2010). Functional and morphological reproductive aspects in male rats exposed to di-n-butyl phthalate (DBP) in utero and during lactation. *J. Toxicol. Environ. Health A.* 73 972–984. 10.1080/15287391003751760 20563931

[B44] Scott H. M. Hutchison G. R. Jobling M. S. McKinnell C. Drake A. J. Sharpe R. M. (2008). Relationship between androgen action in the “male programming window,” fetal sertoli cell number, and adult testis size in the rat. *Endocrinology* 149 5280–5287. 10.1210/en.2008-0413 18566125

[B45] Smirnova A. Mentor A. Ranefall P. Bornehag C. G. Brunström B. Mattsson A. (2021). Increased apoptosis, reduced Wnt/β-catenin signaling, and altered tail development in zebrafish embryos exposed to a human-relevant chemical mixture. *Chemosphere* 264:128467. 10.1016/j.chemosphere.2020.128467 33032226

[B46] Takemura S. Kayama T. Kuge A. Ali H. Kokubo Y. Sato S. (2006). Correlation between copper/zinc superoxide dismutase and the proliferation of neural stem cells in aging and following focal cerebral ischemia. *J. Neurosurg.* 104 129–136. 10.3171/jns.2006.104.1.129 16509156

[B47] Tekin K. Arslan P. Cil B. Filazi A. Akcay E. Yurdakok-Dikmen B. (2020). Companion animals get close to the toxic aspects of antropogenic world: cytotoxicity of phthalates and bisphenol A on dog testicular primary cells. *Cytotechnology* 72 629–638. 10.1007/s10616-020-00401-y 32435861 PMC7547924

[B48] Trovalusci E. Rossato M. Gamba P. Midrio P. (2020). Testicular function and sexuality in adult patients with anorectal malformation. *J. Pediatr. Surg.* 55 1839–1845. 10.1016/j.jpedsurg.2019.12.028 32057441

[B49] Tsutsumi T. Ichihara T. Kawabe M. Yoshino H. Asamoto M. Suzuki S. (2004). Renal toxicity induced by folic acid is associated with the enhancement of male reproductive toxicity of di(n-butyl)phthalate in rats. *Reprod. Toxicol.* 18 35–42. 10.1016/j.reprotox.2003.08.004 15013062

[B50] Venturelli A. C. Meyer K. B. Fischer S. V. Kita D. H. Philipsen R. A. Morais R. N. (2019). Effects of in utero and lactational exposure to phthalates on reproductive development and glycemic homeostasis in rats. *Toxicology* 421 30–40. 10.1016/j.tox.2019.03.008 30940548

[B51] Wang H. Zhou W. Zhang J. Li H. (2019). Role of JNK and ERK1/2 MAPK signaling pathway in testicular injury of rats induced by di-N-butyl-phthalate (DBP). *Biol. Res.* 52:41. 10.1186/s40659-019-0248-1 31387634 PMC6685163

[B52] Zhang T.-D. Zhang L.-D. Xu L.-L. Ma Y.-B. Li H.-C. Wang Z.-M. (2020). Ultrastructural changes in fetal rat testes after mono(2-ethylhexyl)phthalate exposure in vitro. *Int. J. Clin. Exp. Med.* 13 3989–3999.

[B53] Zhang Y. Jiang X. Chen B. (2004). Reproductive and developmental toxicity in F1 Sprague-Dawley male rats exposed to di-n-butyl phthalate in utero and during lactation and determination of its NOAEL. *Reprod. Toxicol.* 18 669–676. 10.1016/j.reprotox.2004.04.009 15219629

