# Supplementary material for: Exposure to Dibutyl Phthalate and Reproductive-Related Outcomes in Animal Models: Evidence From Rodents Study
Source: Front Physiol. 2021 Dec 8;12:684532. doi: 10.3389/fphys.2021.684532 (PMC8692859; doi:10.3389/fphys.2021.684532)
Supplement: Supplementary file 3 [file Table_2.docx]

| 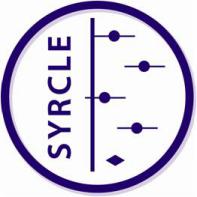 **Systematic Review Protocol for Animal Intervention Studies**  **Format by SYRCLE ([www.syrcle.nl](http://www.syrcle.nl))**  **Version 2.0 (December 2014)** | | | | |
| --- | --- | --- | --- | --- |
| **Item #** | **Section/Subsection/Item** | **Description** | | **Check for approval** |
|  | A. General | | | |
| 1. | Title of the review |  | |  |
| 2. | Authors (names, affiliations, contributions) |  | |  |
| 3. | Other contributors (names, affiliations, contributions) |  | |  |
| 4. | Contact person + e-mail address |  | |  |
| 5. | Funding sources/sponsors |  | |  |
| 6. | Conflicts of interest |  | |  |
| 7. | Date and location of protocol registration |  | |  |
| 8. | Registration number (if applicable) |  | |  |
| 9. | Stage of review at time of registration |  | |  |
|  | B. Objectives | | | |
|  | Background | | | |
| 10. | What is already known about this disease/model/intervention? Why is it important to do this review? |  | |  |
|  | Research question | | | |
| 11. | Specify the disease/health problem of interest |  | |  |
| 12. | Specify the population/species studied |  | |  |
| 13. | Specify the intervention/exposure |  | |  |
| 14. | Specify the control population |  | |  |
| 15. | Specify the outcome measures |  | |  |
| 16. | State your research question (based on items 11-15) |  | |  |
|  | C. Methods | | | |
|  | Search and study identification | | | |
| 17. | Identify literature databases to search (*e.g.* Pubmed, Embase, Web of science) | □MEDLINE via PubMed □Web of Science  □SCOPUS □EMBASE  □Other, namely:  □Specific journal(s), namely: | |  |
| 18. | Define electronic search strategies (*e.g.* use the [step by step search guide](http://www.ncbi.nlm.nih.gov/pmc/articles/PMC3265183/pdf/LA-11-087.pdf)^[15](http://www.ncbi.nlm.nih.gov/pmc/articles/PMC3265183/pdf/LA-11-087.pdf)^and animal search filters^[20,](http://www.ncbi.nlm.nih.gov/pmc/articles/PMC3104815/pdf/LA-09-117.pdf) [21](http://lan.sagepub.com/content/48/1/88.full.pdf+html)^) | When available, please add a supplementary file containing your search strategy: [insert file name] | |  |
| 19. | Identify other sources for study identification | □Reference lists of included studies □Books  □Reference lists of relevant reviews  □Conference proceedings, namely:  □Contacting authors/ organisations, namely:  □Other, namely: | |  |
| 20. | Define search strategy for these other sources |  | |  |
|  | Study selection | | | |
| 21. | Define screening phases (*e.g.* pre-screening based on title/abstract, full text screening, both) |  | |  |
| 22. | Specify (a) the number of reviewers per screening phase and (b) how discrepancies will be resolved |  | |  |
|  | *Define all inclusion and exclusion criteria based on:* | | | |
| 23. | Type of study (design) | Inclusion criteria:  Exclusion criteria: | |  |
| 24. | Type of animals/population (*e.g.* age, gender, disease model) | Inclusion criteria:  Exclusion criteria: | |  |
| 25. | Type of intervention (*e.g.* dosage, timing, frequency) | Inclusion criteria:  Exclusion criteria: | |  |
| 26. | Outcome measures | Inclusion criteria:  Exclusion criteria: | |  |
| 27. | Language restrictions | Inclusion criteria:  Exclusion criteria: | |  |
| 28. | Publication date restrictions | Inclusion criteria:  Exclusion criteria: | |  |
| 29. | Other | Inclusion criteria:  Exclusion criteria: | |  |
| 30. | Sort and prioritize your exclusion criteria per selection phase | Selection phase:  1.  2.  etc.  Selection phase:  1.  2.  etc. | |  |
|  | Study characteristics to be extracted (for assessment of external validity, reporting quality) | | | |
| 31. | Study ID (*e.g.* authors, year) |  | |  |
| 32. | Study design characteristics (*e.g.* experimental groups, number of animals) |  | |  |
| 33. | Animal model characteristics (*e.g.* species, gender, disease induction) |  | |  |
| 34. | Intervention characteristics (*e.g.* intervention, timing, duration) |  | |  |
| 35. | Outcome measures |  | |  |
| 36. | Other (*e.g.* drop-outs) |  | |  |
|  | Assessment risk of bias (internal validity) or study quality | | | |
| 37. | Specify (a) the number of reviewers assessing the risk of bias/study quality in each study and (b) how discrepancies will be resolved |  | |  |
| 38. | Define criteria to assess (a) the internal validity of included studies (*e.g.* selection, performance, detection and attrition bias) and/or (b) other study quality measures (*e.g.* reporting quality, power) | □By use of [SYRCLE's Risk of Bias tool](http://www.biomedcentral.com/1471-2288/14/43/abstract)^[4](http://www.biomedcentral.com/1471-2288/14/43/abstract)^  □By use of SYRCLE’s Risk of Bias tool, adapted as follows:  □By use of [CAMARADES' study quality checklist, e.g](http://www.ncbi.nlm.nih.gov/pubmed/15060322) ^[22](http://www.ncbi.nlm.nih.gov/pubmed/15060322)^  □By use of CAMARADES' study quality checklist, adapted as follows:  □Other criteria, namely: | |  |
|  | Collection of outcome data | | | |
| 39. | For each outcome measure, define the type of data to be extracted (*e.g.* continuous/dichotomous, unit of measurement) |  | |  |
| 40. | Methods for data extraction/retrieval (*e.g.* first extraction from graphs using a digital screen ruler, then contacting authors) |  | |  |
| 41. | Specify (a) the number of reviewers extracting data and (b) how discrepancies will be resolved |  | |  |
|  | Data analysis/synthesis | | | |
| 42. | Specify (per outcome measure) how you are planning to combine/compare the data (*e.g.* descriptive summary, meta-analysis) |  | |  |
| 43. | Specify (per outcome measure) how it will be decided whether a meta-analysis will be performed |  | |  |
|  | *If a meta-analysis seems feasible/sensible, specify (for each outcome measure):* | | | |
| 44. | The effect measure to be used (*e.g.* mean difference, standardized mean difference, risk ratio, odds ratio) |  | |  |
| 45. | The statistical model of analysis (*e.g.* random or fixed effects model) |  | |  |
| 46. | The statistical methods to assess heterogeneity (*e.g.* I^2^, Q) |  | |  |
| 47. | Which study characteristics will be examined as potential source of heterogeneity (subgroup analysis) |  | |  |
| 48. | Any sensitivity analyses you propose to perform |  | |  |
| 49. | Other details meta-analysis (*e.g.* correction for multiple testing, correction for multiple use of control group) |  | |  |
| 50. | The method for assessment of publication bias |  | |  |
|  | | | | |
| Final approval by (names, affiliations): | |  | Date: | |
